# Electroencephalogram-Electromyogram Functional Coupling and Delay Time Change Based on Motor Task Performance

**DOI:** 10.3390/s21134380

**Published:** 2021-06-26

**Authors:** Nyi Nyi Tun, Fumiya Sanuki, Keiji Iramina

**Affiliations:** 1 Graduate School of Information Science and Electrical Engineering, Kyushu University, 744 Motooka, Nishi-ku, Fukuoka 819-0395, Japan; 2 Graduate School of Systems Life Sciences, Kyushu University, 744 Motooka, Nishi-ku, Fukuoka 819-0395, Japan; sanuki.fumiya.547@s.kyushu-u.ac.jp; 3 Faulty of Information Science and Electrical Engineering, Kyushu University, 744 Motooka, Nishi-ku, Fukuoka 819-0395, Japan

**Keywords:** cortico-muscular coherence, electroencephalogram, electromyogram, functional coupling, mutual information, motor task performance, delay time

## Abstract

Synchronous correlation brain and muscle oscillations during motor task execution is termed as functional coupling. Functional coupling between two signals appears with a delay time which can be used to infer the directionality of information flow. Functional coupling of brain and muscle depends on the type of muscle contraction and motor task performance. Although there have been many studies of functional coupling with types of muscle contraction and force level, there has been a lack of investigation with various motor task performances. Motor task types play an essential role that can reflect the amount of functional interaction. Thus, we examined functional coupling under four different motor tasks: real movement, intention, motor imagery and movement observation tasks. We explored interaction of two signals with linear and nonlinear information flow. The aim of this study is to investigate the synchronization between brain and muscle signals in terms of functional coupling and delay time. The results proved that brain–muscle functional coupling and delay time change according to motor tasks. Quick synchronization of localized cortical activity and motor unit firing causes good functional coupling and this can lead to short delay time to oscillate between signals. Signals can flow with bidirectionality between efferent and afferent pathways.

## 1. Introduction

Functional coupling of brain and muscle signals usually appear during voluntary movement. Investigating the coupling between brain and muscle signals is important for rehabilitation of stroke patients and future brain–computer interface (BCI) technology [1]. Functional coupling of brain and muscle signals is usually calculated by using cortico-muscular coherence (CMC). CMC has been recognized as a potential biomarker for recovery from stroke [2,3,4,5]. It is a technique for measuring the strength of correlations between two signals in the frequency domain [6]. The real-world applications of functional coupling and CMC have been found in the invention of movement intention detectors, biomedical robotics, prosthetic devices for people with disabilities and amputees, rehabilitation systems for the stroke patients, in the construction of cortical-muscular functional networks and in numerous research studies for investigating EEG/EMG controllers with different kinds of classifiers etc.

Concerned with functional coupling of brain and muscle signals, previous studies have used positron emission tomography (PET), transcranial magnetic stimulation (TMS), functional magnetic resonance imaging (fMRI) and electroencephalogram (EEG) to investigate coherence mechanisms of motor cortex in patients [7,8]. Some studies initially used magnetoencephalography-electromyogram (MEG-EMG) and electrooculography-electromyogram (ECoG-EMG) to report the functional coupling [9,10]. The studies have performed EEG-EMG correlation analysis with and without the neurofeedback experimental paradigm [11,12]. EEG-EMG coherence was calculated with three types of hand movement tasks such as clench fist, wrist flexion and wrist extension by using magnitude squared coherence (MSC) and wavelet coherence [13]. However, this study used only linear correlation methods and lack of studying different frequencies analysis with the adopted SVM classifier. The researchers constructed the cortical-muscular functional network and classified the accuracy of movements with Fisher and artificial neural network. It still needs better optimization methods in order to simplify the proposed functional networks for real-world applications [14]. Although there were well-documented findings of functional coupling with maintained voluntary contraction, executed precision-pinch tasks, static isometric contraction tasks, wrist flexion and extension tasks with different classifiers, EEG-EMG functional coupling with various motor tasks still needs to be studied to achieve reliable evidence for real-world applications.

One of the major concerns about functional coupling is band frequencies. During motor task performance, cognitive brain signals produce alpha (8–12 Hz) and beta (13–30 Hz) waves and muscle activities produce beta (13–30 Hz) and piper (30–60 Hz) rhythms [15,16]. Coherence occurs in the beta band ranges of both low (13–21 Hz) and high (21–31 Hz) beta in flexor and extensor muscle regardless of contraction [17]. Moreover, studies have concluded that alpha band coherence shows an EMG reflecting ascending or feedback interactions, and gamma band coherence shows an EMG reflecting descending or feedforward interactions [18]. Coherence values of 4–6 Hz and 8–12 Hz are observed when Parkinson’s disease and essential tremor subjects are subjected to the experiment [19,20,21]. Conversely, the coherence value was found to be in the higher beta/low gamma range (30–45 Hz) during dynamic motor tasks [22,23]. Moreover, effects of attention and precision of exerted force can cause beta band EEG-EMG synchronization [24]. The studies indicated that EEG-EMG coherence was detected in the motor imagery condition, while the other studies pointed out that no EMG signals occurred during the motor imagery [3,25,26,27]. Thus, there remains many doubted issues of functional coupling in motor imagery tasks. EMG rectification is one of the problems in coherence analysis. Rectification can cause a significant distortion of the frequency content of an EMG signal [28]. The functional coupling of two signals depends on the specific band frequency ranges, force level, age correlation and use of the rectification process for EMG signals [29,30].

From the perspective of nonlinear correlation analysis, mutual information can provide a measure of nonlinear dependency between two signals [31]. Mutual information can be used to investigate information transmission between EEG-EEG [32,33], delay time and directionality inference between EEG-EMG [27,34]. However, there is a lack of research into the comparison of coupled information across tasks with mutual information methods. The estimation of delay time between two signals can facilitate understanding of the physiology of a given system and information on conduction velocity. Previous studies have indicated that there will be time lag for descending oscillation (from brain to muscle) and ascending oscillation (from muscle to brain) between the sensorimotor cortex and the peripheral muscles [4,35]. The functional coupling of two signals with a delay time usually represents those signals’ propagation time [36,37,38]. As the cortical events propagate to the periphery and the motor cortex also receives input from the periphery, more research is still needed in order to explore the cortico-muscular synchronization from the perspective of delay time with directionality inference [39,40]. Most of the previous studies used the cross correlation method [27] and phase-based methods [34,41] in finding the delay time between two signals; however, a lack of directionality inference was the negative aspect of these methods.

The aim of this proposed study is to fulfill the above unclear facts and remaining problems for functional coupling during different motor tasks. In previous studies, lack of various motor tasks with nonlinear correlation methods, inconsistent occurrence of coherences in different frequency ranges, unjustification of rectified EMG issues and motor imagery condition, attention and inattention effects on coupling level, task-related performances and information flow directionality between brain and muscles were the main motivations of this study and research. Thus, we focused on electroencephalography-electromyogram (EEG-EMG) functional coupling with linear and nonlinear information flow in four different types of motor task condition, such as hand grasping real movement (RM), movement intention (Inten), motor imagery (MI) and movement observation only looking at virtual hand in three dimensional head mounted display (3D-HMD) environment (OL). The abbreviated words RM, Inten, MI and OL for each task were used for the whole discussion of this study. We accounted for the MI and OL tasks together with RM and Inten motor tasks for comparison of functional coupling and delay time as a new experimental task-related perspective based on the state of the art as listed in Appendix A Table A1. To infer directionality of information flow between two signals, we investigated the delay time with the use of lagged power correlation in the specified coupling frequency bands.

The main objective of this study was to investigate the synchronization of brain and muscle signals and coupling delay time that can change based on four different motor tasks. The research facts of this study were aimed to be applied in the rehabilitation systems of stroke patients for clinical applications in future. If patients could move their hand or arm, a correlation of brain and muscle signals might be observed. Then, we can deduce the amount of delay time for that information flow to judge whether the patients recover from stroke level or not during the rehabilitation period. Furthermore, this study evidence can be used in the design of movement intention detectors with various classifiers and prosthetic devices for people with disabilities. Thus, this study proved that EEG-EMG functional coupling and delay time change based on the motor task performance as a preliminary study. The remainder of this paper is organized as follows. Section 2 presents experimental materials in detail. Section 3 discusses methods of the study. Section 4 describes the results. Section 5 presents the discussion. Section 6 provides the conclusions.

## 2. Materials

### 2.1. Participants

This experiment comprised a total of 13 participants who were right-handed. All participants were from Kyushu University and ranged in the age from 21 to 28 years (23.92 ± 1.754 years, mean ± SD). Among the 13 participants, two were females and eleven were males. None of the participants had a physical disorder or brain damage in the past. The study was conducted in accordance with the ethical principles of Kyushu University and the Declaration of Helsinki. The participants provided written informed consent before the experiment.

### 2.2. Experimental Setting

We used g.USBamp of g.tec medical engineering company to record the EEG and EMG signals. Ten EEG channels and three surface EMG (sEMG) channels were used. EEG electrodes were Fp1, Fp2, Cz, FC3, C3, CP3, FC4, C4, CP4, and Pz. Bipolar sEMG electrodes were put on the brachioradialis muscle, flexor carpi ulnaris muscle and flexor carpi radialis muscle, respectively. We recorded both EEG and EMG signals with 1200 Hz sampling rate. All electrodes’ impedance values were under 1 kΩ. To suppress the power line noise interferences, the notch filter 60 Hz was used. The A1 electrode was set as a reference and AFz was set as a ground. In this experiment, we used Oculus company’s oculus rift head mounted display, HMD to make a virtual reality environment. We made the virtual reality environment by using Unity (2019.2.9f1) software and designed a place mimicking a real experimental room in a three-dimensional head mounted display (3D-HMD). We created hand models with MakeHuman software and Blender software for task instructions. After making a file of recording movement, we used this file as an input to the Unity which played this file by using trigger. We used two computers in this experiment. One computer was used for signal recording and the other one was used for making a virtual reality environment.

### 2.3. Experimental Design and Procedures

**Figure 1:**
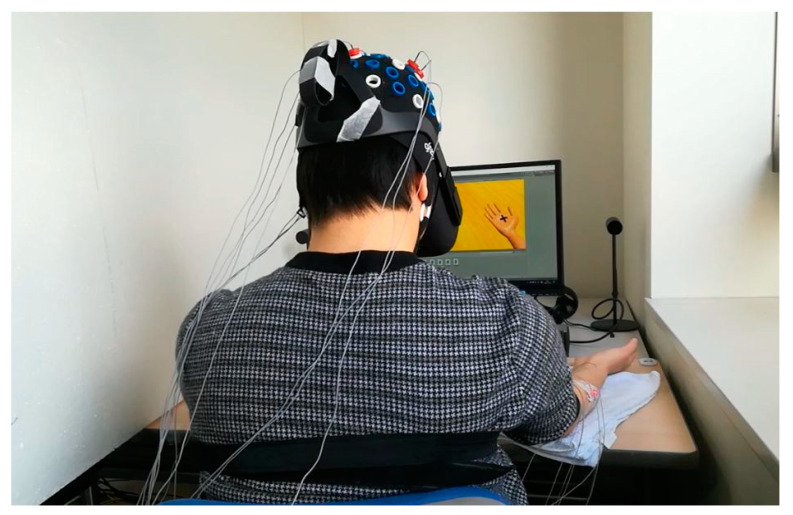
Experimental design for motor task performance using the 3D-HMD condition in the VR environment.

We displayed the created hand models in virtual reality by using head mounted display, HMD for motor task instructions and motor learning of hand grasping tasks. We asked the participants to put both hands on the table in the same position of hand in a virtual reality environment. We placed the towel under the participant’s hand in order not to include force. To reduce physiological artifacts, we asked the participant not to blink, clenching their jaw or make unnecessary movements during recording. Firstly, we demonstrated the motor tasks presented in the work before data acquisition to acclimatize participants with the setup. Then, the instructions for the tasks were shown on the monitor screen via head mounted display, HMD in a virtual reality environment. Figure 1 shows the experimental design.

**Figure 2:**
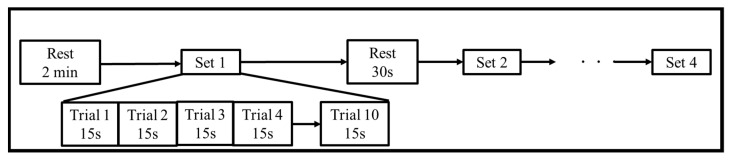
Experimental task flow.

**Figure 3:**
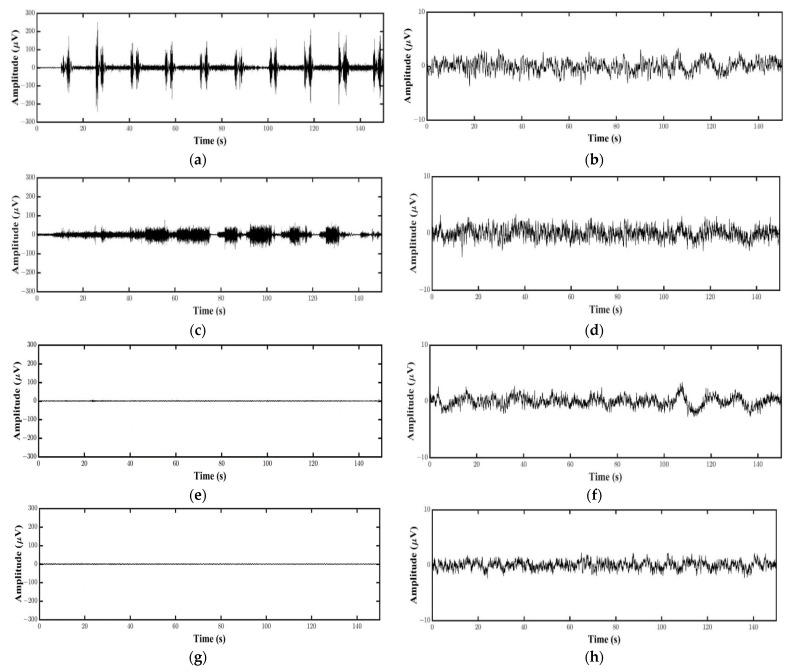
Ten trials of one subject’s EEG and EMG data: (**a**) EMG data in RM task; (**b**) EEG data in RM task; (**c**) EMG data in Inten task; (**d**) EEG data in Inten task; (**e**) EMG data in MI task; (**f**) EEG data in MI task; (**g**) EMG data in OL task; (**h**) EEG data in OL task.

We used four different motor tasks. RM is a task in which a participant moves his or her dominant hand in real-hand grasping movement. Inten is a kind of isometric contraction that involves the static contraction of a muscle without any visible movement in the angle of the joint. MI is a task in which participants carried out a mental process by rehearsing or simulating a given motor action. OL is a task in which participants just looked at virtual hand’s movement without any brain imaging. To ensure the absence of bias, we designed the motor task with four patterns: Inten→OL→RM→MI, OL→RM→MI→Inten, RM→MI→Inten→OL, and MI→Inten→OL→RM. The participants performed one pattern randomly selected from these four patterns. Figure 2 shows the task flow of the experiment. There was a 2 min rest period as a baseline. Then, there were 8 s of rest, 2 s of being ready and 5 s of the task in 1 trial. We designed a total of 40 trials with 4 sets in each motor task. A fixation cross was shown on the virtual palm during rest, which disappeared during the 2 s ready stage. The virtual hand grasping appeared on the monitor in HMD during 5 s task. The time to break between each motor task was 5 min, then RM, Inten, MI and OL tasks were performed, respectively. Figure 3 shows the first ten trials data of one subject in each task for both EEG and EMG data.

### 2.4. Data Analysis

Among 10 channels of EEG data, we chose only the contralateral brain motor cortex, C3 as it was mainly concerned with body movement in the brain [13]. Among three EMG channels, we used only flexor carpi ulnaris muscle since this muscle was directly involved in hand grasping movement. Since this study would like to emphasize the coupling and their delay time change based on the tasks and bands, we chose only a single electrode of EEG and EMG at this time. In data preprocessing, we resampled both signals to 256 Hz for reducing computation speed and time. We chose the bandpass filter range to 1 to 100 Hz for both signals. The EEG data that contained artifacts was determined by visual inspection with the use of EEGLAB. We used ICA as it is an effective tool for rejecting several types of non-brain artifacts. To remove eye-blinking and muscle noises, the data that were over the limit ±100 μV were excluded. We rejected at most one or two ICA components that apparently affect the EEG channel data. Then, we extracted EEG data. For EMG signals, the non-rectified EMG signals were filtered with selected bandpass filter and then exported to further analysis [2,9].

For statistical analysis, firstly, we used the Shapiro–Wilk normality test to verify the normality of the data with (*p* > 0.05). We used bootstrap estimation for each ANOVA test as it is an effective method for creating the non-normal data to normality. We further applied the generalized linear model together with one-way ANOVA for achieving more specific information between different variables. The one-way ANOVA was performed based on frequency bands and designed tasks. The normality test for the mutual information showed a non-normal distribution with (*p* < 0.05). The Kruskal–Wallis test was used to compare more than two groups in the nonparametric method. For the delay time comparison of the beta and gamma bands, one-way ANOVA was performed after conducting the Shapiro–Wilk test for normality. We used LSD and Bonferroni correction methods for all pairwise comparisons with *p* < 0.05. IBM SPSS 20 (SPSS Inc., Chicago, IL, USA) was used for all statistical comparisons.

## 3. Methods

### 3.1. Functional Coupling of Brain and Muscle Signals with Linear Correlation Analysis

Coherence is the principal measure of linear correlation between two signals in the frequency domain. The range of coherence is between zero and one, where one indicates a perfect linear relationship and zero indicates two signals that are not linearly correlated at that frequency.

To determine functional coupling between two signals, we calculated the EEG-EMG coherence for each task. After preprocessing the data, we took only 0–5-s EEG and non-rectified EMG data. For a single trial, we calculated the frequency space relationships between two data sets of EEG and EMG signal with a 19-ms non overlapping Hanning window and a frequency resolution of 2 Hz then all segments were transformed to the frequency domain using the fast Fourier transform. We then computed auto power spectral $S_{\mathrm{xx}}\;$,${\;S}_{\mathrm{yy}}$ and cross power spectral $S_{\mathrm{xy}}$ for both signals. After this, we calculated the coherence for one trial. We calculated coherence values between EEG and EMG at the frequency, f, for every trial and then averaged the data to access the changes in coherence by using Equation (1).

(1) $${\mathrm{Coh}}_{\mathrm{xy}}\left(f\right)={\left|S_{\mathrm{xy}}\left(f\right)\right|}^{2}/S_{\mathrm{xx}}\left(f\right)\times {\;S}_{\mathrm{yy}}\left(f\right)$$

The coherence value significance level was determined based on the confidence limit, CL as in Equation (2).

(2) $$\mathrm{CL}=1\;-{\;(1\;-\;\alpha )\;}^{1⁄\left(L-1\right)}$$

where L represents the number of data segments used in the coherence calculation and α is a confidence interval and is typically 95% [13].

(3) $$A_{\mathrm{coh}}=\underset{f}{\sum }\Delta f({\mathrm{Coh}}_{\mathrm{xy}}\left(f\right)\;-\;\mathrm{CL})$$

For statistical comparison, the coherence areas for each frequency band range were calculated by using the formula as in Equation (3), where Δf represents frequency resolution, f is the frequency of the calculated band, and ${\mathrm{Coh}}_{\mathrm{xy}}\left(f\right)$ is the coherence value.

### 3.2. Functional Coupling of Brain and Muscle Signals with Nonlinear Correlation Analysis

The coherence method is a linear method and it cannot be used for the study of complex and nonlinear brain dynamics. Mutual information is a flexible analysis framework that can be applied to identify the patterns of connectivity regardless of the distributions of the data, for example, linear, nonlinear and circular [36].

To examine the functional coupling with nonlinear correlation analysis, we computed the mutual information between EEG, C3 versus EMG signals during four different motor task types. The data from two electrodes was computed with a sliding 100 ms segment and a step size of 50 ms over all trials in the data range of −2 s to 5 s time series, then we calculated the amount of mutual information between two signals. To calculate the mutual information, the entropy of the signals are required to compute. Thus, we firstly bin the data to create a histogram with Matlab function hist. Next, we compute the probability that a value of the data would fall into each bin. Then, we multiply the probability value by the logarithm-base-2 of that probability value and sum all probability values for entropy [36]. After that, we calculated mutual information between EEG and EMG. We compared the amount of mutual information across all motor tasks by using Equation (4).

(4) $$\mathrm{MI}\left(X,Y\right)=H\left(X\right)+H\left(Y\right)-H\left(X,Y\right)\;=\sum_{j=1}^{m}\sum_{i=1}^{n}p(x_{i},y_{j}){\;\log}_{2}\left[p\left(x_{i},y_{j}\right)/p\left(x_{i}\right)p\left(y_{j}\right)\right]$$

### 3.3. Delay Time Investigation with Nonlinear Mutual Information Flow

The delay time of mutual information can be used to infer the directionality of information flow between two signals [36] (p. 404). The coherence and mutual information of two signals do not appear at the same time but with a little delay time that can tell us the possible information processing of motor tasks and flow directionality of two signals. In previous studies, delay time during functional coupling was investigated with the phase-based method, Hilbert transform method and cross correlation analysis [27,41]. However, these methods limit its applicability to the narrow band coherence and existence of minimal phase relation in two signals and they are not be capable to infer the directionality of information flow. Thus, we investigated the delay time with lagged power correlation in specific frequency bands to infer the direction of information flow between efferent and afferent pathways.

Since the highest coherence values were occurred in the range of beta (13–30 Hz) and gamma (31–50 Hz) in results, we calculated the delay time of EEG and EMG signals in those bands by making power fluctuations time series using Morlet wavelet transformation. We considered two time series X(t) and Y(t) (t = 1…T) at T discrete points [42]. First, we constructed the time-frequency representation of beta and gamma bands based on the Morlet wavelet decomposition, which provides an optimal concentration in time and frequency [34]. Morlet wavelet, ω(t,f) in terms of time and frequency representation is given as in Equation (5).

(5) $$\omega \left(t,f\right)={\;\mathrm{Ae}}^{\left(-t^{2}/2\sigma_{t}^{2}\right)}e^{\left(2i\pi \mathrm{ft}\right)},$$

We calculated the convolution of the wavelet with the signal from the epoch at time instant, t and every frequency, f. The square norm of the convolution was the time varying energy of the both EEG and EMG signals at a specific frequency, as in Equation (6).

(6) $$\;\;\;\;\left|E_{x}\left(t,f\right)\right|={\left|\omega \left(t,f\right){\;*\;x}_{q}\left(t\right)\right|}^{2},$$

We computed mutual information repeatedly for the multiple time lags by taking the shifting one signal with respect to another by using Equation (7). Then, it was graphed by calculating mutual information between two signals by fixing EEG signals and measuring the information according to the delay time in EMG signals [36] (p. 404). If the information present at the location EEG is transmitted to location EMG, there will be a peak in the curve with TDMI(X(t), Y(t + τ)) at τ > 0. If the information present at the location EMG is transmitted to location EEG with delay time TDMI(X(t), Y(t + τ)) at τ < 0. A peak that occurs for τ = 0 implies that a zero delay for the EMG and EEG may be due to the nullification of two strong counteracting forces driven from EEG to EMG and opposing drive from EMG to EEG [37].

(7) $$\mathrm{TDMI}\left(X\left(t\right),Y\left(t+\tau \right)\right)=H\left(X\left(t\right)\right)+H\left(Y\left(t+\tau \right)\right)-H\left(X\left(t\right),Y\left(t+\tau \right)\right)\;\;=\underset{n}{\sum }p\left(x\left(t\right),y\left(t+\tau \right)\right)\times {\;\log}_{2}\left[p\left(x\left(t\right),\;y\left(t+\tau \right)\right)/p\left(x\left(t\right),y\left(t+\tau \right)\right)\right]$$

## 4. Results

### 4.1. Comparison of EEG-EMG Coherence in Each Motor Task Based on Bands

**Figure 4:**
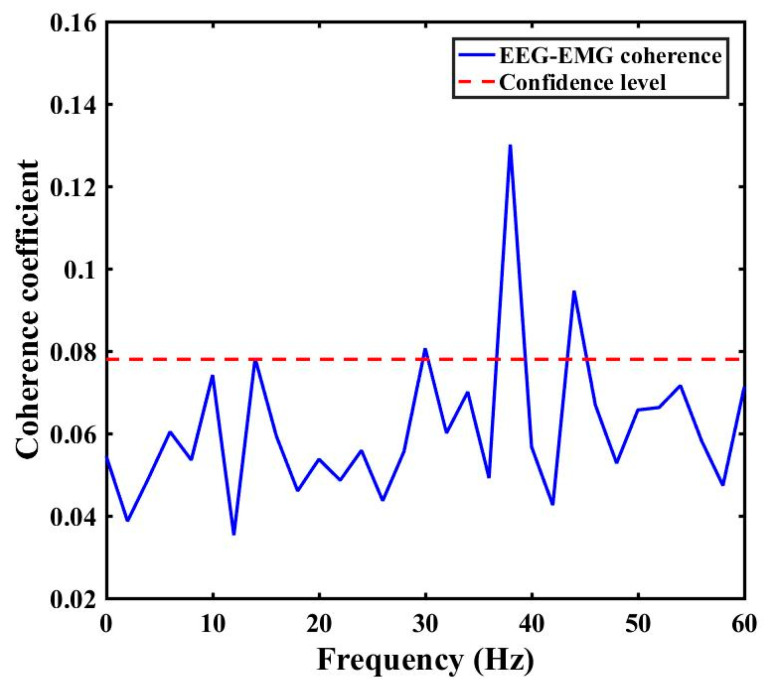
EEG-EMG coherence of one subject data in RM task.

To predict the pattern of coherence clearly, the coherence of one subject’s data in RM task was shown in Figure 4. The highest coherence appeared at ~38 Hz during the task. As we had already mentioned above, the functional coupling can happen in different specified frequency bands within different muscle contraction types. Thus, we firstly checked the averaged values of coherence during coupling in delta (0.5–3.5 Hz), theta (4–7.5 Hz), alpha (8–12 Hz), beta (13–30 Hz) and gamma (31–50 Hz) ranges for all motor tasks. The first hypothesis was that the functional coupling of brain and muscle signals in five different bands was not significantly different in each RM, Inten, MI and OL tasks.

We used the Shapiro–Wilk normality test to check the normality distribution in the statistical analysis. All the motor tasks showed normality with *p* > 0.05 in all bands. Then, we performed a one-way ANOVA test with bootstrap estimation and generalized linear model for the comparison of coherence based on bands in each motor task. The results rejected the null hypothesis and showed a significant difference among all band groups with one-way ANOVA (F(4,60) = 3.159, *p* = 0.02, $\eta_{p}^{2}=0.174$) in the RM task. The LSD post-hoc test showed a significant difference of coherence (mean ± SE) between the alpha band (0.0503 ± 0.0029, *p* = 0.018) and beta band (0.0618 ± 0.0018). There was also significant difference between the delta band (0.0549 ± 0.0050, *p* = 0.047), theta band (0.0534 ± 0.0043, *p* = 0.022) and alpha band (0.0503 ± 0.0029, *p* = 0.004) compared to the gamma band (0.0645 ± 0.0012).

The coherences in Inten task also rejected the null hypothesis with a significant difference one-way ANOVA (F(4,60) = 4.578, *p* = 0.003, $\eta_{p}^{2}=0.234$). The LSD post-hoc test resulted in a significant difference of coherence (mean ± SE) between the delta band (0.0540 ± 0.0030, *p* = 0.029), theta band (0.0543 ± 0.0018, *p* = 0.036), and alpha band (0.0522 ± 0.0023, *p* = 0.007) compared to the beta band (0.61162 ± 0.0018). There was also a significant difference among the delta band (0.0540 ± 0.0030, *p* = 0.006), theta band (0.0543 ± 0.0018, *p* = 0.008), and alpha band (0.0522 ± 0.0023, *p* = 0.001) compared to the gamma band (0.0631 ± 0.0019).

**Figure 5:**
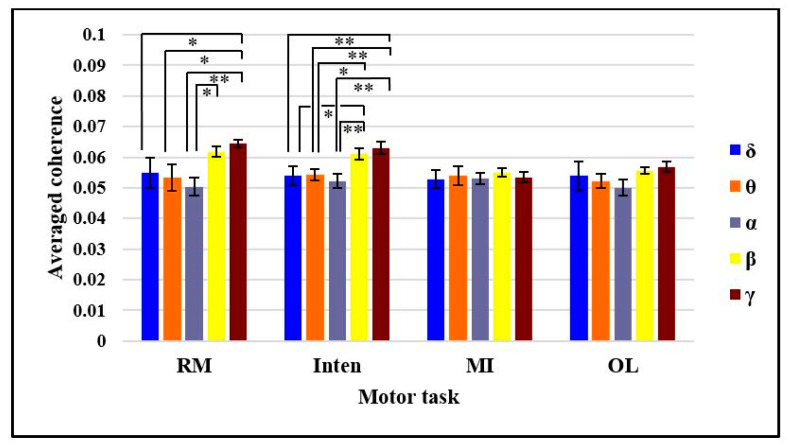
Comparison of the averaged coherence based on frequency band in four motor tasks. Error bars show the standard error of the mean. * *p* < 0.05 ** *p* < 0.01.

However, there was no significant difference among the five different bands with one-way ANOVA (F(4,60) = 0.140, *p* = 0.967,$\;\eta_{p}^{2}=0.009$) in the MI task and (F(4,60) = 0.926, *p* = 0.455, $\eta_{p}^{2}=0.058$) in the OL task, as shown in Figure 5. Thus, Bonferroni correction post-hoc tests were used for multiple comparisons of MI and OL tasks. According to the results, we could say that the functional coupling of two signals can occur in all types of band but with different amounts. The highest coherences appeared in beta and gamma bands of RM and Inten tasks while all five bands had low coherences in MI and OL tasks.

### 4.2. Comparison of EEG-EMG Coherence in Beta Band and Gamma Bands Based on Motor Tasks

As the higher coherences were detected in the beta and gamma bands, we selected these two bands among five bands and then compared the averaged coherence again based on the tasks in all subjects. Thus, we hypothesized again that functional coupling coherence between cortex and muscle in beta and gamma bands was not statistically significantly different across four types of motor tasks.

**Figure 6:**
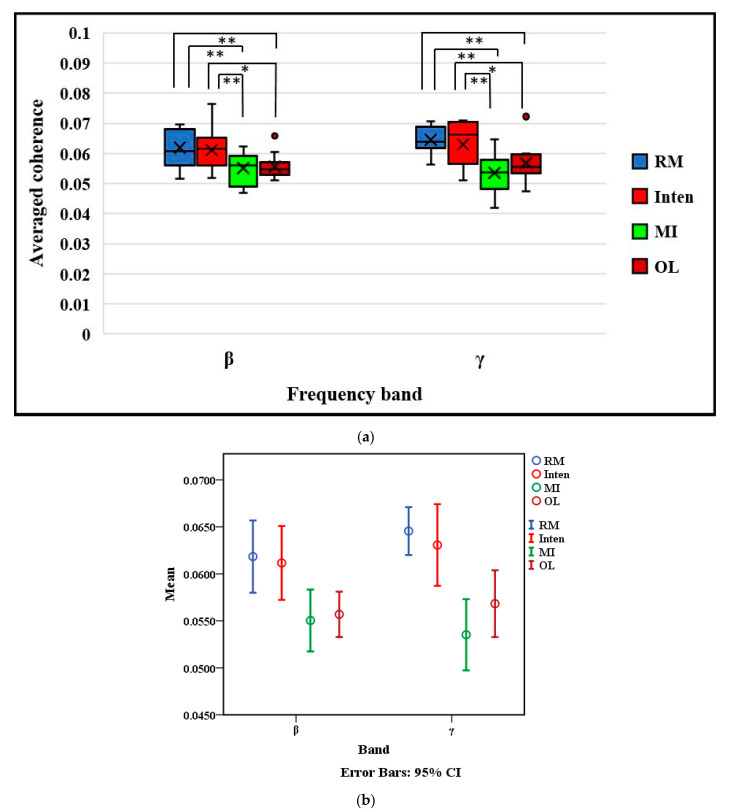
Comparison of the averaged coherence in beta band and gamma band based on motor tasks: (**a**) The top and bottom of each box represent the 25th and 75th percentiles, respectively. Cross sign inside each box represents the mean value. The horizontal black line represents the median. The whiskers are drawn from the ends of the interquartile ranges to the minimum and maximum values. * *p* < 0.05 ** *p* < 0.01. (**b**) Circle marked points represent the means and bars of these points represent the 95% CI of the within-subject standard error.

However, the results also rejected the null hypothesis. The higher coherence occurred in only RM and Inten tasks in both band ranges, as shown in Figure 6a. In the beta band, we could clearly observe the averaged coherence amount of RM, Inten, MI and OL tasks with a significantly different task × coherence value of (F(3,48) = 5.145, *p* = 0.004, $\eta_{p}^{2}=0.243$) in the ANOVA test. The LSD post-hoc test showed a significant difference of coherence (mean ± SE) between the MI task (0.0550 ± 0.0015, *p* = 0.004) and OL task (0.0557 ± 0.0011, *p* = 0.008) compared to the RM task (0.0618 ± 0.0017), and between the MI task (0.0550 ± 0.0015, *p* = 0.008) and the OL task (0.0557 ± 0.0011, *p* = 0.017) compared to the Inten task (0.0611 ± 0.0018). There was no statistically significant difference between the RM task and Inten task (*p* = 0.762).

In the gamma band, the result also showed high coherence in the RM and Inten tasks rather than MI and OL tasks, with a task × coherence value of one-way ANOVA(F(3,48) = 9.812, *p* = 0.001, $\eta_{p}^{2}=0.380$). Then, the LSD post-hoc test showed a significant difference between the MI task (0.0535 ± 0.0117, *p* < 0.001) and OL task (0.0568 ± 0.0016, *p* = 0.002) compared to the RM task (0.06455 ± 0.0011) and MI task (0.0535 ± 0.0117, *p* < 0.001) and OL task (0.0568 ± 0.0016, *p* = 0.011) compared to the Inten task (0.0631 ± 0.0199) in the gamma band. As with the beta band, there was no statistically significant difference between the RM task and Inten task in gamma band (*p* = 0.530).

**Figure 7:**
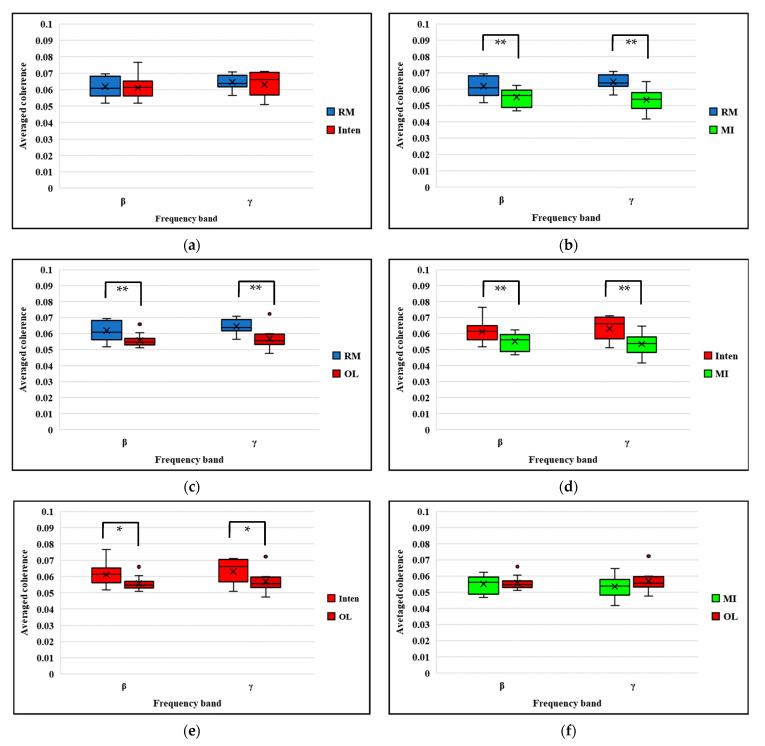
EEG-EMG coherence comparison across all subjects in beta band and gamma band: (**a**) RM task versus Inten task; (**b**) RM task versus MI task; (**c**) RM task versus OL task; (**d**) Inten task versus MI task; (**e**) Inten task versus OL task; (**f**) MI task versus OL task. * *p* < 0.05 ** *p* < 0.01.

To be able to check the individual level of the independent variables and to look at the confidence interval in terms of true mean values for coherence, we also performed the 95% CI of the within-subject standard error estimation of coherences across the tasks in both beta and gamma bands as shown in Figure 6b. Finally, to evaluate the magnitude and variability of coherence across tasks, we further constructed box and whisker plots which depicted the mean coherence obtained within two frequency bands of beta and gamma, as shown in Figure 7. The changed coherence in all subjects was compared across each task. The results confirmed that the functional coupling or interaction between brain and muscle signals can be greater in the RM and Inten tasks rather than the other MI and OL tasks if two signals synchronized well during the motor tasks execution. These results confirmed again that the functional coupling of EEG-EMG change based on motor task performance.

### 4.3. Comparison of EEG-EMG Mutual Information Based on Motor Tasks

**Figure 8:**
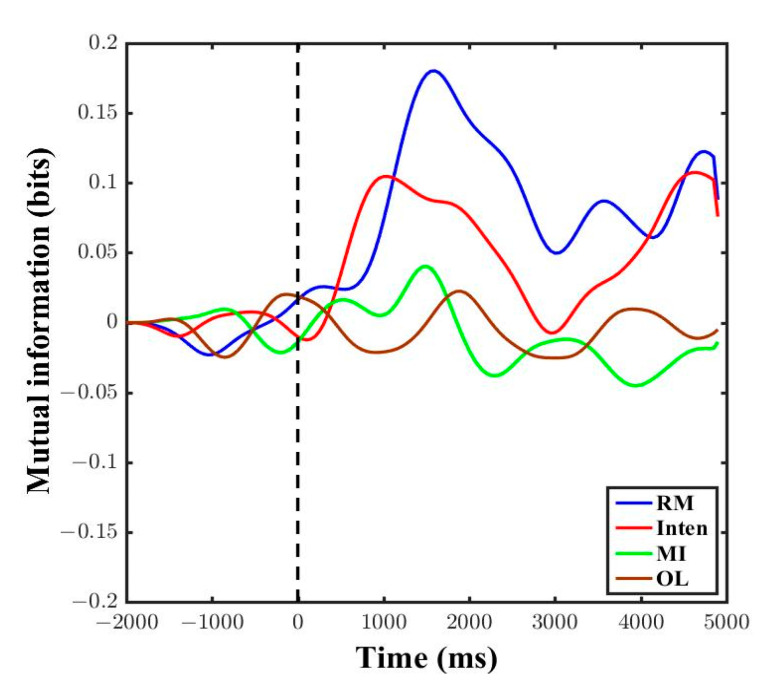
Comparison of mutual information in time series of data from one subject across all motor task conditions. The black vertical dotted line represents the point at which the participant was given the motor task instructions.

Since coherence is a linear method and it cannot be applied for the complex brain and muscle signals, we checked the mutual information across all four kinds of motor task performance. Figure 8 shows the result of mutual information amount in one subject. The amount of information in the RM task increased during the 5 s task after instructions began. The Inten task also showed a greater amount of mutual information during the 5 s motor task as nearly the same result of RM. However, the amount of mutual information was low during the MI and OL tasks. There were small fluctuations concerned with the subjects’ motor tasks preparation and learning before the stimulus time point. The results showed that there was very low functional coupling between brain and muscle signals if there was no actual movement and intention to move. The nonlinear mutual information could prove that functional coupling amounts vary across the types of motor task.

### 4.4. Comparison EEG-EMG Averaged Mutual Information Across All Subjects

**Figure 9:**
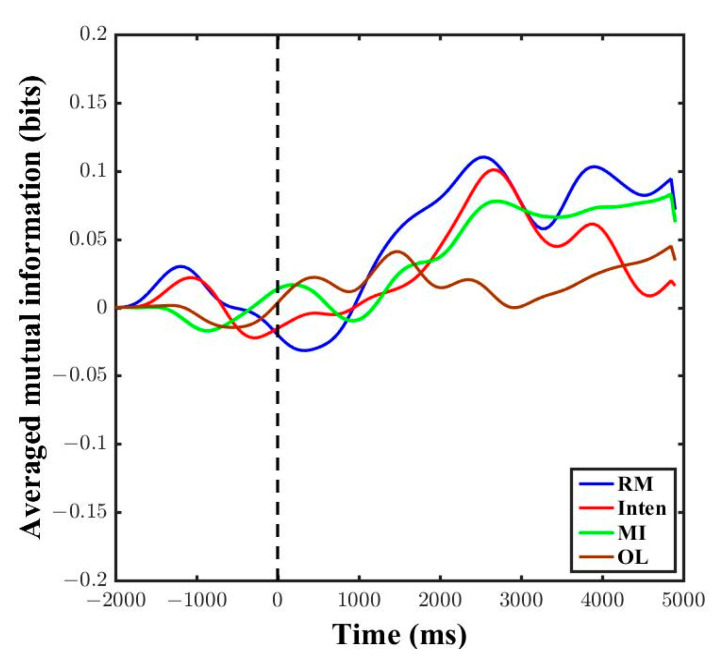
Comparison of mutual information in time series of data from all subjects across all motor task conditions. The black vertical dotted line represents the point at which the participants were given the motor task instructions.

We calculated the averaged mutual information across all subjects. In the results, the RM showed the highest amount of mutual information during the 5 s motor task, then the Inten task followed, with the second highest amount of information between brain and muscle signals. Then, the MI and OL tasks showed a slight increase in mutual information in averaged data, and this might concern the subjects achieving focused attention and sensory motor integration after some period of stimulus, as shown in Figure 9. In addition, there were some fluctuations for movement preparation before the subjects performed the tasks.

**Figure 10:**
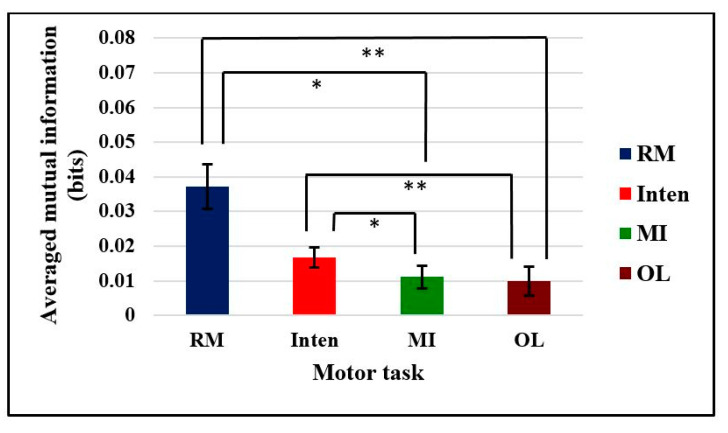
Averaged mutual information comparison across all motor tasks. The asymptotic significance (two-sided tests) is displayed with a standard error bar. The significant differences in each motor task were * *p* < 0.05 ** *p* < 0.01.

For statistical analysis, the further null hypothesis was that functional coupling between brain and muscle signals was not different across motor tasks in nonlinear mutual information. We took only the mean absolute value of mutual information from the 0–5 s data. According to the Shapiro–Wilk normality distribution test, the results showed that mutual information data were not distributed with *p* < 0.05. Thus, we had used the nonparametric Kruskal–Wallis test and independent-sample Kruskal–Wallis test for multiple group comparisons. We rejected the null hypothesis with a significant difference in mutual information across the four motor tasks (Chi square = 16.65, *p* = 0.001, df = 3). There was a significant difference in mutual information (mean ± SE) between the MI task (0.0111 ± 0.0033, *p* = 0.042) and OL task (0.0099 ± 0.0041, *p* < 0.001) compared with the RM task (0.0372 ± 0.0064) and between the MI task (0.0111 ± 0.0033, *p* = 0.023) and OL task (0.0099 ± 0.0041, *p* = 0.002) compared with the Inten task (0.0167 ± 0.0029), as shown in Figure 10. There was no statistically significant difference between RM task and Inten task (*p* = 0.230). We could say that the more synchronized the cortical neurons and motor firing units inside the cell during tasks, the higher the functional coupling between brain and muscle signals. Thus, the greater amount of functional coupling between two signals totally depend on the motor tasks.

### 4.5. Calculation Delay Time between Brain Motor Cortex and Peripheral Muscle

**Figure 11:**
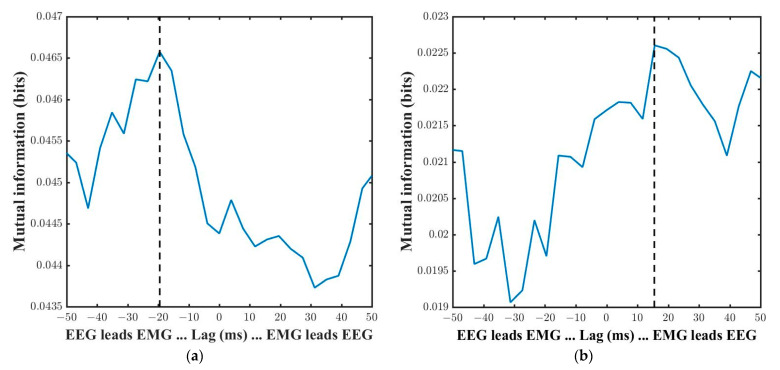
Delay time mutual information of one subject in RM task: (**a**) in beta band; (**b**) in gamma band. The black vertical dotted line represents the delay time at the maximum value of mutual information.

We finally investigated whether a good correlation of two signals would require a smaller delay time in signal transmission from one to each other or not. Based on the occurrence of high coherence within the frequency ranges of beta (13–30 Hz) and gamma (31–50 Hz), the delay time values were calculated to determine the signal propagation and interaction time from the motor cortex to the muscle periphery or the muscle periphery to the motor cortex. In the calculation, a positive value of delay indicates that the time series of EMG is in advance; a negative value of delay indicates that the time series of EEG is in advance [36,38]. The results of delay time mutual information in one subject data for the beta and gamma bands of RM task was shown in Figure 11. Based on the results, we could infer that information flow direction with (−)20-ms lagged time from peripheral muscle to motor cortex in beta band and (+)15-ms lagged time from cortex to muscle in gamma band.

We reported delay time values of each subject across all motor tasks in both beta and gamma ranges in Table 1. According to the results, the averaged delay time in the RM and Inten tasks was in the range from 15–25 ms, in agreement with [4]. Conversely, the delay time in the no movement tasks, such as the MI and OL conditions, were higher than those in the RM task and Inten task in both beta and gamma bands. The amount of delay time in the gamma band also occurred with a smaller amount of mutual information if we compared it with the beta band. It is noteworthy that the higher the frequency ranges we investigated, the lower the delay time, with lower amount of mutual information resulted. This might have occurred in the gamma band ranges of averaged results for all subjects. Some subjects showed a zero delay time. A zero delay might be due to the nullification of strong counteracting forces drive from motor cortex to muscle and opposing drive from muscle to motor cortex. This result indicates that the amount of time to transmit the signals from one area to another could be high if they were not coherent or even if they had a low correlation. In summary, good functional coupling of brain and muscle would require a smaller delay time for signal transmission in both efferent and afferent pathways.

### 4.6. Statistical Analysis of Delay Time across All Subjects

We also performed a statistical analysis of the averaged delay time to check multiple comparisons across all subjects in four kinds of motor task. First, we checked the normality distribution of the data with the Shapiro–Wilk test. The data of each task for all individuals together showed normal distribution with *p* > 0.05. Then, we used the parametric test ANOVA with the task × delay time value for statistical analysis. A significant difference was found among four different motor tasks in the beta band delay time with one-way ANOVA(F(3,48) = 8.479, *p* = 0.001, $\eta_{p}^{2}=0.361$). The LSD post-hoc test showed a significant difference among the MI task (33.38 ± 1.5129 ms, *p* < 0.001) and OL task (35.31 ± 2.32 ms, *p* < 0.001) compared with the RM task (22.77 ± 1.31 ms), and between the MI task (33.38 ± 1.5129 ms, *p* = 0.011) and OL task (35.31 ± 2.32 ms, *p* < 0.001) compared with the Inten task (25 ± 2.71 ms). There was no statistically significant difference between the RM task and Inten task beta band delay time (*p* = 0.445).

**Figure 12:**
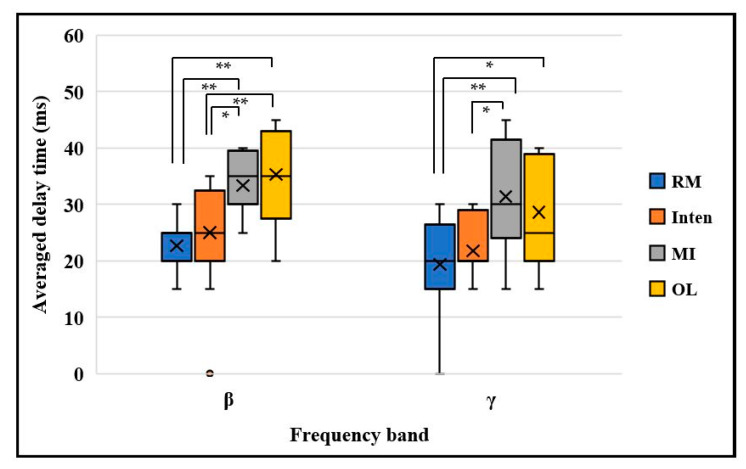
Averaged delay time mutual information comparison across all motor tasks in beta band and gamma band. The top and bottom of each box represent the 25th and 75th percentiles, respectively. The cross sign inside each box represents the mean value. The horizontal black line represents the median. The whiskers are drawn from the ends of the interquartile ranges to the minimum and maximum values. * *p* < 0.05 ** *p* < 0.01.

In the gamma band delay time, the results also showed a significant difference with (F(3,48) = 4.053, *p* = 0.012, $\eta_{p}^{2}=0.253$). The LSD post-hoc test resulted in the MI task (31.46 ± 2.71 ms, *p* = 0.003) and OL task (28.61 ± 2.46 ms, *p* = 0.012) compared with RM task, and the MI task (31.46 ± 2.71 ms *p* = 0.044) compared with Inten task (21.77 ± 2.262 ms), as shown in Figure 12. We could use this to prove that the EEG-EMG functional coupling delay time values in both beta band and gamma band are also significantly different and variable based on the motor task performance.

## 5. Discussion

### 5.1. EEG-EMG Functional Coupling Analysis Using Linear Coherence

Using the previous studies on cortical-muscular coherence and functional network, this study hypothesized that EEG-EMG functional coupling changes based on different motor task performance [3,13,14,43]. The results proved that the functional coupling between brain and muscle signals varies depending on the motor tasks that subjects executed. In this study, coupling amounts were greater in the RM and Inten tasks than in the other MI and OL tasks in all subjects. Functional coupling of EEG-EMG signals was systematically decreased and enhanced at specific frequencies of interest from 0.5 Hz to 50 Hz across the four motor tasks. Our results satisfied the remaining controversial issue of previous studies concerned with band-specific problems, namely whether the highest coherences can appear only in the beta band and gamma band or not. Both beta and gamma bands can appear in RM and Inten tasks, while all five bands had low coherences in MI and OL tasks.

The high coherence values depict that there is strong physiological underpinning as an indicator of neural binding across the tasks [44]. From a physiological perspective, RM, Inten, MI and OL tasks require different patterns of coordination among cortical and motor neurons to produce the necessary motions and forces. Thus, this study motivated the four tasks since they are distinct from the perspective of mechanical requirements such as force and motion etc. These fundamental mechanical differences were also among one of the phenomena that brain and muscle activity coordination changes associated with tasks.

Based on the occurrence of highest coherence in beta and gamma bands during clench fist, wrist flexion and extension tasks, the study extracted features and applied a SVM classifier for reclassifying a motor task among three different motor tasks [13]. Next, the study constructed the cortical-muscular functional network and classified hand movements with Fisher and artificial neural network for exploration of more effective methods in human behavior perception. The researchers applied theta, alpha, beta and gamma frequency bands for their constructed model [14]. Our study confirmed again the same occurrence of beta and gamma bands coherence during the motor tasks execution as the previous studies [13,14].

There were low coherences in delta, theta and alpha bands and high coherences in beta and gamma bands. The results of alpha band coherence is typically thought to reflect the afferent feedback through the stretch reflex loop [44]. There was very low coherence in the alpha band across all tasks in the study. The coherences were more apparent in the efferent pathways than afferent feedback. The higher band coherences are typically thought to reflect cortical drive to muscles. Beta band coherence is thought to be very sensitive to movement. It usually occurred during the maintenance of static and isometric force [10,15,22]. In addition, the occurrence of coherence in the beta band could be related to the ERD/ERS phenomenon as an interactive effect of it [45]. The firing behavior of spinal motoneurones and cortical activity correlated as a functional coupling within the beta ranges [9]. The study concluded that the amount of significant beta range synchronization decreases below the confidence level when the attention is divided between motor tasks and other simultaneously performed tasks. The cortical-muscular network works in good synchronization when the attentive resources are directed towards the motor tasks. Beta range EEG-EMG synchronization was the effect of attention and precision of exerted force during a maintained motor contraction task [24].

The integration of visual and somatosensory information increment could shift cortico-muscular coherence to the gamma range [22]. The 40 Hz rhythms could have occurred during motor preparation and controlling of finger movement performance [46]. Neuronal gamma band (40–70 Hz) coherence has been found along the visuomotor pathways and is concerned with visuomotor interactions [21,47]. In this study, we applied 3D-HMD for the motor task commands and stimulation for the subjects. This process could lead to a focused attention from the participants and produced sensory motor integration of brain signals and then resulted in the gamma range (31–50 Hz) coherences [24]. Cortical gamma band oscillation may reflect the efferent drive to the muscle during very strong tonic contraction and dynamic forces [15,22,48].

In addition, as we used the 19 ms Hanning windowing in coherence analysis, this would effectively create a ~50 Hz high pass of the original signal. However, this high-pass filter effect of this derivation can remove the activity with low spatial frequencies, including volume conducting activity. Thus, coherent values in high frequency beta and gamma bands cannot be due to volume conduction, and resulting coherence values are purely as a consequence of the execution of different motor tasks. The functional coupling of higher bands’ results were consistent with the results reported in [23,49]. The coherences could occur in both bands without using special dynamic forces in our experiment.

Coherence similarities between cortical activities occurred during the imaginary neuromuscular activities [3,25,26]. Nevertheless, there was very low coherence in the MI and OL tasks, except for some subjects in the OL task. In summary, our results showed that the coupling ranges totally change based on the motor task performance, as we had already discussed. The expression and gating of coherent discrete cortical and spinal networks during motor tasks may be a mechanism to appear as good functional coupling between two signals.

### 5.2. EEG-EMG Functional Coupling Analysis Using Nonlinear Mutual Information

The study used the wavelet coherence and magnitude squared coherence (MSC) to calculate the EEG-EMG coherence based on hand movements and to classify the movements with SVM classifier [13]. However, there has been a lack of studies with nonlinear correlation methods for the construction of cortical-muscular functional networks with different types of classifiers [14]. This study was the initial study with nonlinear information flow calculation across different task conditions.

Comparison of mutual information across different motor tasks was one of the essential state of the art requirements in brain–muscle correlation, as in Appendix A Table A1. Thus, we extended our study to fill the gap of functional coupling with different motor tasks. When the averaged mutual information was investigated in accordance with the different motor tasks, a greater amount of mutual information was found during the RM and Inten tasks than during the MI and OL tasks. The information increased starting from the baseline onset zero point as shown in Figure 8 and Figure 9. Using a 100 ms sliding window in analysis can create an effective high pass filter and this can lead to achieving the pure task data mutual information. Increased mutual information of RM and Inten tasks revealed that there were good coupled signals between the brain and muscle signals during motor task execution. The absence of good synchronization between two signals could lead to a small amount of mutual information. The nonlinear mutual information results were also consistent with the linear coherence method in this study.

In addition, we observed that the MI and OL tasks showed a slight increment after the task instructions point in averaged mutual information results. This transient increase of mutual information during motor imagery and movement observation might be the influences and consequences of subjects’ attention and the impulse responses of visual stimulation [21,47]. These occurrences were the same coincidence as the occurrence of higher gamma band coherence, caused by visual effects in linear analysis [25]. Thus, the mutual information results could determine whether there is a good relationship or not in the form of functional coupling across motor tasks.

During hand grasping tasks, motor unit firing and cortical neuron burst inside the cell and caused synchronization, and finally appeared as an action, which we had already proven. In [33], the authors used schizophrenic patients and then checked information transmission between different cortical areas by estimating the average cross mutual information (AMI), but they only used brain signals. The authors used DTF based on the MVAR and AR models, but there were still limitations for linear dependencies [50,51,52]. Thus, we applied both EEG and EMG signals to further explore the relationships between brain and muscle signals by applying the nonlinear information gain method. In summary, this nonlinear correlation method also totally proved that EEG-EMG functional coupling of brain and muscle signals change based on the motor task performance.

### 5.3. Functional Coupling Delay Time Change Based on Motor Tasks

Delay time calculation is fundamentally important for brain–muscle interaction, especially in the design of prosthetic devices and movement intention detectors. The previous studies used only classifiers, and then classified the movement types and did not calculate the amount of the time lag between brain and muscle signals [14,53]. Thus, we finally emphasized the calculation of EEG-EMG functional coupling delay time based on the motor task performance. It is well-known that the direction of information flow cannot be calculated by the functional coupling of coherence. The conventional mutual information is also limited in that it cannot be used for the direction of information flow because it is a symmetric measure. To overcome this limitation, we used delay time mutual information by defining a time series in one of the variables to calculate mutual information, which can lead to an asymmetric measure. The delay time between EEG and EMG data was 11–27 ms between the tremor correlated parts (cortex) of the brain EEG and the trembling hand EMG. The coherence delay time was calculated based on the highest coherence frequency bands as a function of time lag, but the authors could not infer the directionality of information flow [38].

Based on the nerve fibers’ conduction velocity of 50–65 m/s in the arms and the distance between the scalp and the hand of approximately 1.2 m, most delay times are in the range from 18–24 ms [54]. We chose the beta (13–30 Hz) and gamma (31–50 Hz) ranges since there has been a lack of delay time analysis based on these bands. Our results indicate that the averaged delay time values were within the range of 15–25 ms for RM and Inten tasks. These ranges were consistent with physiological facts, as we discussed above [4,54]. Moreover, there was a longer delay time in the no movement task condition of the MI and OL tasks. These results showed that the time will take longer or higher for the transmission of signals from one point to another if there is no high coupling or greater mutual information. The gamma band delay time averaged values showed a smaller amount of delay time than the beta band delay time averaged values.

The advantage of mutual information time lag was that we could infer the direction of information flow based on the polarity of the time value rather than the linear method of coherence. Thus, we could clearly see the signal propagation or transmission time from brain to muscle (descending) or muscle to brain (ascending) oscillation in terms of the lag time, as in Table 1. Some subjects showed information flow from brain to peripheral muscle, and some subjects showed information flow from peripheral muscle to brain [35,55]. In our research, investigation of the delay time with the nonlinear method based on the beta and gamma bands represented a new approach with directionality inference. However, future studies still need to be undertaken in order to obtain more exact results with more subjects with different ages. In summary, EEG-EMG functional coupling delay time values are also significantly different and they change based on the motor tasks in both beta and gamma bands according to the results.

### 5.4. Real-World Applications, Limitations and Future Works of Study

From the perspective of real-world situations, the resulting facts and evidence of this study are aimed to be able to apply in the rehabilitation systems for training stroke patients in future. We can decide the physiological and anatomy changes of patients based on the data of functional coupling level of brain and muscles during training period [2,12]. However, it needs to be tested with a more optimized experimental model. In addition to stroke rehabilitation systems, this research can be applied in the study of human motion and movement for behavioral science such as sports activities, root cause of fatigue, cortical-muscular functional network studies, treatment of dyskinesia and Alzheimer disease and recognition of human motion intention for movement intention detectors with various classifiers etc. [14]. Our study is the updated study of functional coupling with delay time in beta and gamma bands that can be helpful in judging the response time of brain–muscle signals in patients and in construction of motion intention detectors [13,14]. This research has the benefit of many real-world applications in daily life in all possible ways. Furthermore, the results of the current study can give stronger arguments for both previous studies and current studies of cortical-muscular coupling in neuroscience fields.

In calculation of the linear coherence method, there are some important notes and limitations. The output results of coherence depend on the windowing and selected filter design. Thus, suitable window and filter ranges must be chosen in order to achieve the correct results. Next, the analyzed data need to be cleared artifacts as far as it can be. In some cases, EMG signals may become the noise for EEG. Thus, we need to choose the suitable ICA components during preprocessing. For the calculation of nonlinear correlation, the selection of the bin number is also somewhat complex to obtain the optimized values for entropy. Mutual information calculation can be quite difficult if the data are non-stationary. Thus, data needs to be stationary for the calculation of correlation between two signals. It is necessary to make sure that the data are roughly equally noisy across all conditions, electrode pairs and subjects groups. Using surface EMG (sEMG) signals may impact the calculation of signal correlation. Contamination of signals from the neighboring muscles can cause cross talks in data recording. Thus, the surface EMG (sEMG) has some limitations, in comparison to using fine wire EMG electrodes [56].

This new experimental paradigm might lead to future investigations such as a hypothesis regarding whether the gamma band in the OL task might relate to visual stimulation, visuomotor pathways and attention or not. The number of subjects participating in this study were also small and we need to test with more participants of different ages, real stroke patients, control subjects and control tasks to obtain an absence of bias. Different types of visual stimulations and feedback paradigms still need to be investigated in order to explore the effect of attention and visualization on the information processing during functional coupling [24]. Additionally finding out the effect of motor imagery with different experiments of kinesthetic and visual for EEG-EMG coupling is also required. Functional coupling investigation with different types of movement features and classifiers are important for movement detectors, prosthetic devices and controllers for real-world applications [13,14,53]. Construction of a brain–muscle functional network in terms of nonlinear and delay time methods is one of the problems to be explored in future. To establish the directionality of information flow precisely, it still needs to be investigated with transfer entropy, DTF, granger causality and other directionality inference methods.

## 6. Conclusions

This study fulfilled the requirement of a functional coupling study with different motor task conditions that have not been performed in much of the existing literature. Depending on the motor tasks executed by the participants, the functional coupling amount and delay time varied. The results proved that the cortical muscle coupling levels were high only in the beta and gamma bands, and not in the other three bands during the tasks. The beta and gamma band frequencies do not highly depend on force levels, according to the results. In addition, this research demonstrates that a high correlation and association between two signals occurred when the participants performed the motor tasks of RM and Inten. The results of the Inten task coupling level were almost the same as those of the RM task and it was a peculiar and innovative result for almost all subjects. The new consideration of the MI and OL motor tasks together with the RM and Inten tasks confirmed that the unclear controversial issues to be cleared, since low signal correlations occurred in those two tasks. However, as an exception, some subjects showed a slightly high correlation in the OL task. Thus, the new interesting evidence to study in future will be whether the attention caused by the OL task will lead to the high coupling of brain and muscle signals or not, and how it benefits the coupling system. Finally, we explored the signal propagation delay time with directionality inference. The information can flow with exact amount of delay time from efferent to afferent and afferent to efferent pathways when coupling exists. In conclusion, we proved that functional coupling between motor cortex and muscle was statistically different in all delta, theta, alpha, beta and gamma bands based on the tasks. Thus, this research showed EEG-EMG functional coupling and their delay time change according to the task-related performance.

## Figures and Tables

**Table 1:** Summary of the delay time in the beta band and gamma band across all motor tasks.

Delay Time (ms) Obtained by Maximizing Mutual Information ^1^								
Subject	RM		Inten		MI		OL	
	β	γ	β	γ	β	γ	β	γ
1	−20	+15	+20	−20	−40	−15	−45	−30
2	−20	+15	+20	−20	−30	−20	−45	−39
3	25	+20	+25	−20	+25	−45	35	+35
4	+30	+23	−25	+25	+30	−28	+35	−40
5	+25	−30	0	−30	−35	−30	+20	−39
6	−20	+30	−35	−15	−25	−30	−43	−25
7	−25	−20	−35	−20	−40	+20	−43	+20
8	−30	0	−25	−20	−30	+30	−35	−25
9	−15	−15	+30	−25	−40	−28	−35	−20
10	−25	−30	+15	−30	−35	−35	−25	+25
11	−16	+23	−35	−28	−39	−43	−43	−39
12	−20	−15	−30	+30	+35	−40	+25	−20
13	+25	−15	−30	0	−30	−45	−30	−15
Mean	22.76	19.31	25.00	21.76	33.38	31.46	35.31	28.61
SD	4.83	8.35	9.78	8.16	5.45	9.78	8.37	8.86

^1^ Delay time values were calculated by maximizing the mutual information for the thirteen subjects. Positive and negative signs were introduced to infer the directionality of information flow and these polarities were not taken into account in calculation.

## Data Availability

Not applicable.

## Appendix A

**Table A1:** Comparison table for different methods of the state of the art in functional coupling of EEG and EMG.

Ref.	Authors	Investigated Area	Method	Strengths and Weakness
[3]	Yasunari, H., et al., 2010	EEG-EMG coherence during Isometric contraction and its imagery.	Power spectrum. EEG—Cz, FCz, C1, C2, CPz. Rectified EMG—right TA muscle. n = 13	Coherence occurred in motor imagery conditions. Uses linear correlation analysis. Remains controversial issues of EMG during motor imagery.
[9]	James, M., et al., 2000	Task-dependent modulation in coherence between motor cortex and hand muscles.	Amplitude and phase correlation method. MEG—over the left sensorimotor cortex. EMG—1DI, AbPB, FDS, EDC. n = 13	Tests task-dependent modulation of coherence. Coherence was much lower level during isometric grip of the fixed levers compared to grasp under complaint conditions. Remains to investigate with different motor tasks.
[10]	Shinji, O., et al., 2000	ECoG-EMG coherence during isometric contraction in hand muscle.	Auto spectra and frequency domain analysis method. Rectified EMG. ECoG—mesial and lateral surfaces of frontoparietal cortices. EMG—ECR muscle. n = 8 (patients with epilepsy)	Coherence occurred only in the 15 ± 3 Hz beta bands. Time lags were calculated with a cross-correlogram method. Time lags range from 10 ms to 22 ms. Lack of directionality inference and nonlinear correlation. Remains to find out the coherence in other bands.
[22]	Wolfgang, O., et al., 2006	Gamma range Cortico-muscular coherence during dynamic performance in visuo motor tasks.	Cortico motor spectral power method. EEG—52 electrodes. Rectified EMG—flexor digitorum superficialis muscle. n = 8	Beta band coherences occur during static force. Gamma band coherence occurs during dynamic force. Uses only linear correlation method. No include delay time estimation. Remains task-dependent CMC investigation.
[34]	Seung-Hyun, P., et al., 2010	Linear and nonlinear information flow with time delay mutual information.	Used surrogate data sets and experimental data sets. Investigated CM interaction during a right wrist extension tasks. EEG—29 electrodes. EMG—Extensor digitorum communis. n = 7	Well-distinguished linear and nonlinear information flow. Requires relatively long stationary time series data for the analysis. Needs to improve directionality inferences with stationarity.
[41]	Andreas, W., et al., 2012	Time delay mutual information of the phase as a measure of functional connectivity.	Phase lag index and weighted phase lag index methods. Making numerical implementation. Synthetic data sets by a mutual amplitude coupled network of Rossler oscillatior.	Limitations and assumptions existed as synthetic data sets were applied. De-correlation step does not respect a background synchronicity. Uses a data-driven approach.
